# Infarction or Metabolic Breakdown? Longitudinally Extensive Diffusion-Restricted Lesions from the Medulla Oblongata to the Lumbar Spinal Cord

**DOI:** 10.3390/diagnostics16030504

**Published:** 2026-02-06

**Authors:** Yuka Nakaya, Koji Hayashi, Mamiko Sato, Yohei Midori, Toyoaki Miura, Hiromi Hayashi, Kouji Hayashi, Yasutaka Kobayashi

**Affiliations:** 1 Department of Rehabilitation Medicine, Fukui General Hospital, 55-16-1 Egami-cho, Fukui 910-8561, Japansatomoko@f-gh.jp (M.S.);; 2 Graduate School of Health Science, Fukui Health Science University, 55-13-1 Egami, Fukui 910-3190, Japan; khayashi@fukui-hsu.ac.jp (K.H.); yasutaka_k@fukui-hsu.ac.jp (Y.K.); 3 Department of Gastroenterology, Fukui General Hospital, 55-16-1 Egami-cho, Fukui 910-8561, Japan

**Keywords:** infarction, diffusion magnetic resonance imaging, myelopathy, ornithine carbamoyltransferase deficiency disease, methotrexate, demyelinating diseases, copper deficiencies, case report

## Abstract

A 78-year-old woman with a history of rheumatoid arthritis (treated with methotrexate) developed disturbed consciousness, emesis, and intestinal perforation. Initial labs revealed hyperammonemia (189 μg/dL) and hypertonic dehydration. Despite ammonia normalization, her neurological status improved only slightly, necessitating additional tests. Cerebrospinal fluid analysis showed no pleocytosis but positive oligoclonal bands and markedly elevated myelin basic protein (>500 pg/mL). Serum autoimmune markers were negative, including anti-aquaporin-4 (AQP4), anti-myelin oligodendrocyte glycoprotein (MOG), and anti-glial fibrillary acidic protein (GFAP) antibodies. MRI revealed T2/DWI-hyperintense lesions in the left parietal lobe and cerebellum. Crucially, extensive T2/DWI-hyperintense lesions with diffusion restriction spanned the white matter from the medulla oblongata to the lumbar spinal cord. Axial spinal DWI demonstrated diffuse hyperintensity throughout the entire white matter, accompanied by gray matter atrophy. Subsequent metabolic screening revealed low folate and hypocupremia (34 μg/dL) as well as urinary orotic acid and low serum citrulline, suggesting late-onset ornithine transcarbamylase (OTC) deficiency. Given the clinical context, this was interpreted as a metabolic breakdown rather than an established genetic diagnosis. This case is characterized by a long, diffusion-restricted lesion from the brainstem to the spinal cord that does not correspond to vascular territories. She experienced sudden death. We hypothesize that an underlying metabolic disorder, nutritional deficiencies and drug-induced neurotoxicity contributed to lesion formation.

**Figure 1:**
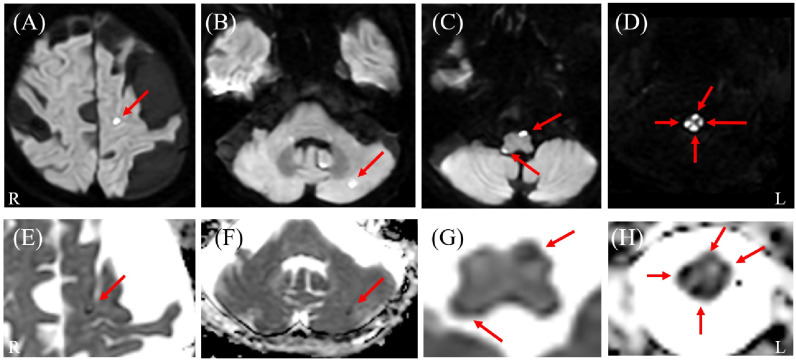
Brain magnetic resonance imaging (MRI) results. Diffusion-weighted imaging (DWI) (**A**–**D**) and corresponding apparent diffusion coefficient (ADC) maps (**E**–**H**) reveal multiple scattered lesions with diffusion restriction in the cerebral hemispheres, cerebellum, and brainstem (arrows). Notably, the brainstem lesion extends longitudinally and is continuous with the spinal cord involvement. L: left; R: right.

**Figure 2:**
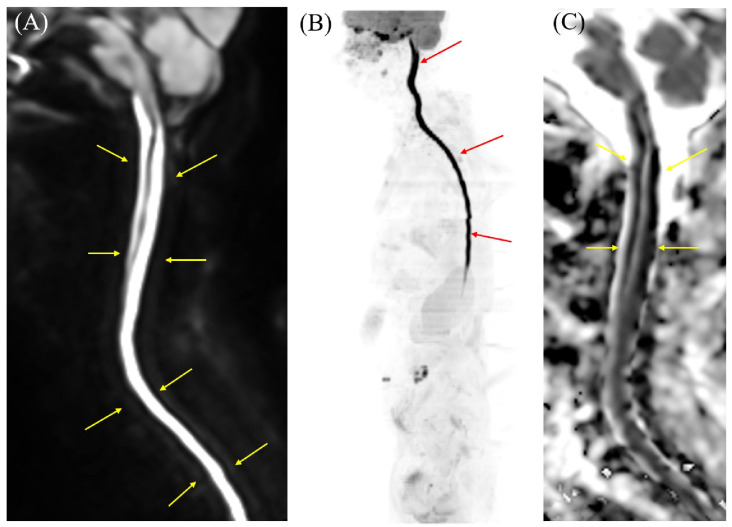
Cervicothoracic MRI on DWI, diffusion-weighted whole-body imaging with background body signal (DWIBS), and ADC map results. (**A**) DWI demonstrates longitudinal hyperintensity extending from the cervical to the lumbar spinal cord (arrows). Similarly to the intracranial findings, this lesion demonstrated restricted diffusion. (**B**) DWIBS vividly displays high signal intensity along the entire cervicothoracolumbar spinal cord (arrows). DWIBS, first described by Takahara et al. in 2004 [1,2], is a sophisticated MRI technique rooted in DWI, which probes tissue microstructure via water molecule Brownian motion [2,3]. Employing short tau inversion recovery (STIR) echo-planar imaging (EPI) [1,2,3], it applies fat suppression and intense diffusion weighting to mute signals from healthy tissues, generating a stark “black background” [2,3]. Regions of restricted diffusion—e.g., malignancies, abscesses, or acute inflammation—stand out as bright hyperintensities [1,2,3,4,5]. These can be rendered as 3D PET-like images and co-registered with T2-weighted sequences for superior anatomical correlation [2,3]. (**C**) ADC maps show low ADC values corresponding to the areas of high signal intensity on DWI (arrows).

**Figure 3:**
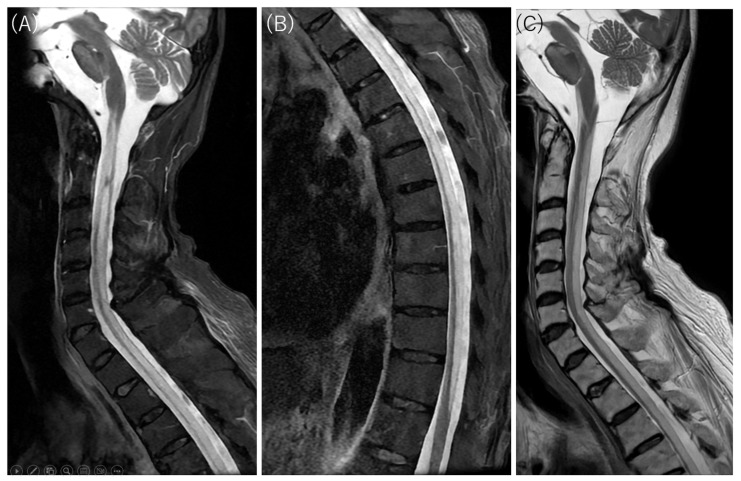
Cervicothoracic MRI findings. Hyperintensity on short tau inversion recovery fast spin-echo (STIR FSE) (**A**,**B**) and T2-weighted imaging (**C**) corresponds to the longitudinally extensive diffusion-restricted lesions observed along the spinal cord. These axial sections reveal diffuse involvement of the entire white matter circumference and robust diffusion restriction (low ADC), distinguishing the pathology from the central gray matter target and isointense or increased ADC values typical of neuromyelitis optica spectrum disorder (NMOSD) [6,7]. Although the massive elevation of CSF myelin basic protein (>500 pg/mL) and positive oligoclonal bands indicate an extensive breakdown of the myelin sheath, the diagnosis of NMOSD and other inflammatory myelopathies was excluded by negative serology for anti-aquaporin-4 (AQP4), anti-myelin oligodendrocyte glycoprotein (MOG), and anti-glial fibrillary acidic protein (GFAP) antibodies, combined with a lack of cerebrospinal fluid (CSF) pleocytosis and the clinical attribution of emesis to intestinal perforation rather than an area postrema lesion.

**Figure 4:**
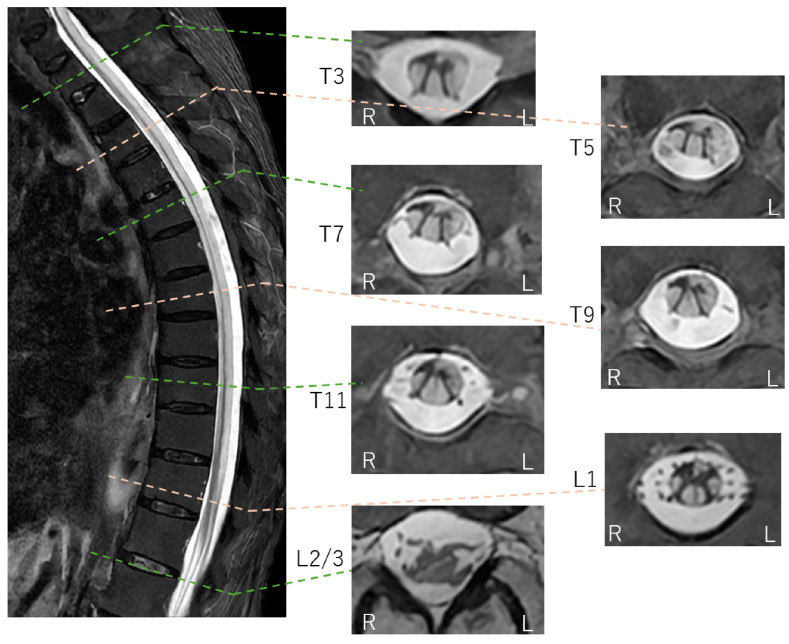
Thoracolumbar STIR FSE MRI findings (axial section). Corresponding to the hyperintensities seen on DWI, high signal intensities are observed within the spinal cord extending down to the lumbar level on STIR-FSE images. In the axial sections, diffuse hyperintensities are noted in the anterior, lateral, and posterior columns. Conversely, the areas with preserved signal intensity are consistent with the spinal gray matter, which appears markedly atrophic. This tract-predominant distribution is a key imaging signature that distinguishes this case from typical spinal cord infarction (SCI), which follows specific arterial territories (anterior or posterior spinal arteries). While venous infarction similarly lacks arterial territory adherence, it is excluded here due to its rarity and the absence of predisposing vascular malformations. Methotrexate (MTX) neurotoxicity is a potential synergistic factor in this case. Although typically associated with intrathecal administration, oral MTX used for the patient’s rheumatoid arthritis can induce white matter vacuolar degeneration [8,9,10]. The observed tract-predominant distribution—specifically involving the posterior and lateral columns—aligns with the known patterns of MTX-induced cord injury, albeit rarely manifesting as such longitudinally extensive involvement from oral dosing. Suspected late-onset ornithine transcarbamylase (OTC) deficiency is supported by hallmark biomarkers, including hyperammonemia, urinary orotic acid, and low citrulline [11,12], though definitive genetic or enzymatic confirmation was not performed. Mechanistically, hyperammonemia-induced glutamine accumulation leads to astrocyte swelling and cytotoxic edema [11,12,13], potentially explaining the diffuse, tract-predominant diffusion restriction observed from the brainstem to the spinal cord. Folate deficiency (potentially due to MTX’s side effect) and hypocupremia (34 μg/dL) might act as synergistic factors in this LESCL, as copper is essential for mitochondrial electron transport and myelin maintenance [14,15,16,17]. Resulting enzymatic dysfunction typically manifests as symmetric T2 hyperintensities in the dorsal and lateral columns [14,15,16,17]. Collectively, the imaging and biochemical profile suggests a catastrophic metabolic breakdown of the spinal white matter architecture driven by the combined effects of suspected late-onset OTC deficiency, hypocupremia, and MTX-induced neurotoxicity. When neuroimaging patterns mismatch recognized vascular or inflammatory territories, complex metabolic and toxic etiologies must be prioritized in the differential diagnosis of LESCL. L: left; R: right.

## Data Availability

The data presented in this study is available on request from the corresponding author. Due to patient privacy and ethical considerations, the data is not publicly accessible.

## Supplementary Materials

The following supporting information can be downloaded at: https://www.mdpi.com/article/10.3390/diagnostics16030504/s1, File S1: The CARE reporting checklist.

## Abbreviations

The following abbreviations are used in this manuscript:

CT	Computed tomography
MRI	Magnetic resonance imaging
DWIBS	Diffusion-weighted whole-body imaging with background body signal suppression
LESCL	Longitudinally extensive spinal cord lesion
MBP	Myelin basic protein
MTX	Methotrexate
OTC	Ornithine transcarbamylase
NMOSD	Neuromyelitis optica spectrum disorder
EPI	Echo-planar imaging
DWI	Diffusion-weighted imaging
ADC	Apparent diffusion coefficient
MOG	Myelin oligodendrocyte glycoprotein
AQP4	Aquaporin-4
GFAP	Glial fibrillary acidic protein
SCI	Spinal cord infarction
