# Supplementary material for: Infarction or Metabolic Breakdown? Longitudinally Extensive Diffusion-Restricted Lesions from the Medulla Oblongata to the Lumbar Spinal Cord
Source: Diagnostics (Basel). 2026 Feb 6;16(3):504. doi: 10.3390/diagnostics16030504 (PMC12896448; doi:10.3390/diagnostics16030504)
Supplement: Supplementary file 1 [file diagnostics-16-00504-s001.zip › diagnostics-4118151-supplementary.pdf]

The CARE reporting checklist

	Item Description	Location (or reason for not reporting)
**Sections**		
1. Title	The area of focus and “case report” should appear in the title.	√ (Interesting Images do not require “Case Report”, but added it in Keyword)
2. Keywords	The key elements of this case in 2–5 words.	√
3. Abstract	3a – Introduction: What does this case add?<br>3b – Case presentation:<br>• The main symptoms of the patient(s).<br>• The main clinical findings.<br>• The main diagnoses and interventions.<br>• The main outcomes.<br>3c – Conclusion: What are the main “take-away” lessons from this case?	√ (Brainstem-to-lumbar white matter involvement)<br>√ (Disturbed consciousness, emesis, hyperammonemia; Suspected OTC deficiency/hypocupremia; sudden death)<br>√ (Metabolic/drug synergy in LESCL formation)
4. Introduction	Brief background summary of the case referencing the relevant medical literature.	√ (Unique tract-specific diffusion restriction)
5a. Patient information	5a – Demographic information of the patient (age, gender, ethnicity, occupation).<br>5b – Main symptoms of the patient (chief complaint).<br>5c – Medical, family, and psychosocial history—including lifestyle and genetic information whenever possible, details about relevant comorbidities, and past interv...	√ (78-year-old woman)<br>√ (Disturbed consciousness, emesis, intestinal perforation)<br>√ (History of RA/MTX; family history was unreported)
6. Clinical findings	Describe the relevant physical examination (PE) findings.	√ (Slight neurological improvement despite NH3 normalization)
7. Timeline	Depict important date and times in this case (table or figure).	No (Interesting Images do not require Timeline.)
8. Diagnostic assessment	8a – Diagnostic methods (e.g., physical examination, laboratory testing, imaging, questionnaires)<br>8b – Diagnostic challenges (e.g., financial, language, or cultural)	√ (Metabolic screen, CSF (OCB/MBP), DWI/ADC/DWIBS)<br>√ (Slight improvement despite normalizing labs)

	8c – Diagnostic reasoning including other diagnoses considered<br><br>8d – Prognostic characteristics (e.g., staging) where applicable.	✓ (Suspected OTC deficiency/hypocupremia; excluded NMOSD/SCI)<br><br>✓ (Sudden death occurred)<br><br>In Abstract and Figure 3/4 legends
9. Therapeutic Intervention	9a – Types of intervention (e.g., pharmacologic, surgical, preventive, self-care)<br><br>9b – Administration (e.g., dosage, strength, duration)<br><br>9c – Changes in intervention (with rationale).	No (Focus is on diagnosis; specific new therapy not detailed)
10. Follow up and outcomes	10a – Clinician and patient-assessed outcomes<br><br>10b – Important follow-up test results (positive and negative)<br><br>10c – Intervention adherence and tolerability (and how this was assessed)<br><br>10d – Adverse and unanticipated events.	✓ (Slight neurological improvement then sudden death)<br><br>✓ (Metabolic screening (orotic acid, citrulline))<br>No (Not applicable due to death.)<br><br>✓ (Sudden death)
11. Discussion	Discussion (including conclusion):<br><br>11a – Strengths and limitations of the management of this case<br><br>11b – Relevant medical literature<br><br>11c – Rationale for conclusions (including assessment of cause and effect)<br><br>11d – Main “take-away” lessons of this case report.	✓ (Strength: multi-modal imaging; Limit: genetic confirmation)<br><br>✓ (OTC, hypocupremia, MTX neurotoxicity discussed)<br><br>✓ (Catastrophic metabolic breakdown hypothesis)<br><br>✓ (Necessity of metabolic/toxic screening in LESCL)<br>In Figure 3 and 4 legends
12. Patient perspective	When appropriate patients should share their perspectives on the treatments they received.	No (Not possible; consent obtained from family)
13. Informed consent	Did the patient give informed consent? Please provide if requested.	No (Not possible; consent obtained from family)
